# Supplementary material for: LEAFY demonstrates functions in reproductive development of the gametophyte but not the sporophyte of the fern Ceratopteris richardii
Source: Development. 2026 Jan 2;153(1):dev204808. doi: 10.1242/dev.204808 (PMC12813671; doi:10.1242/dev.204808)
Supplement: Supplementary information [file develop-153-204808-s1.pdf]

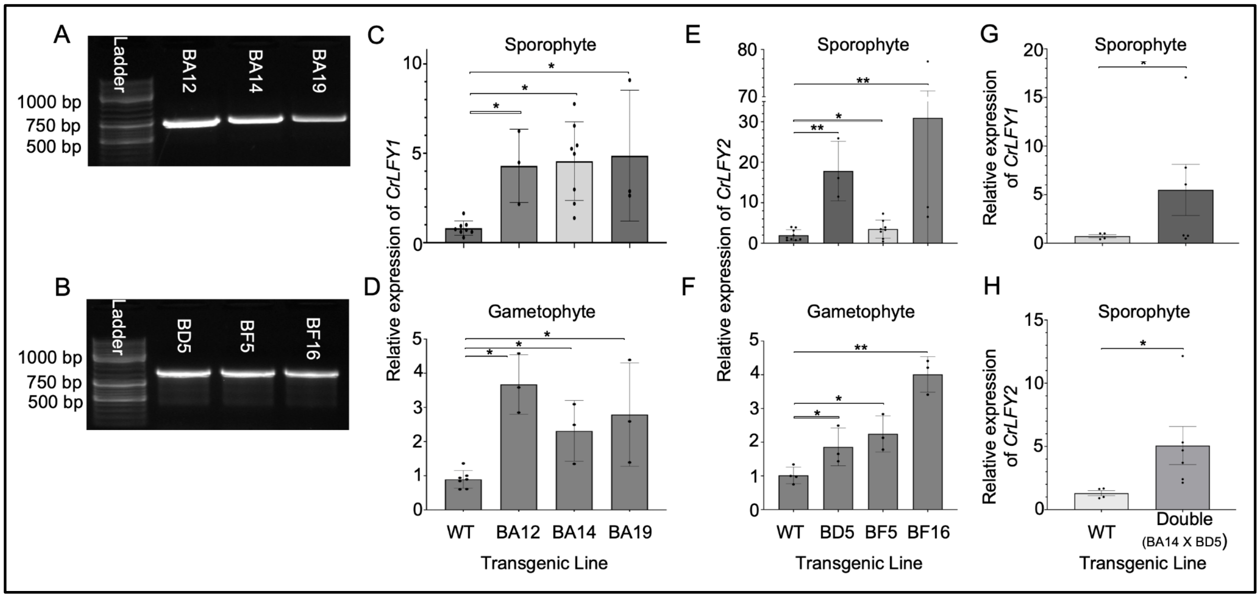

Fig. S1. Molecular and expression validation of transgenic plants in this study. (A) gDNA amplification of three independent transgenic lines per construct characterized here; primers specific for the *35S::CrLFY1* cassette showing bands of the expected size (753 bp). (B) Same for *35S::CrLFY2* transgenic lines (824 bp). (C-D) Relative expression of *CrLFY1* to the housekeeping genes *CrACT1* and *CrTBPb* comparing wild type (WT) to three independent *35S::CrLFY1* transgenic lines characterized in this study, for (C) sporophytes or (D) gametophytes. (E) Relative expression of *CrLFY2* in WT and *35S::CrLFY2* for (E) sporophytes and (F) gametophytes. (G-H) *CrLFY1* and *CrLFY2* expression in the double transgenic line. Expression of *CrLFY1/2* was significantly higher in transgenic plants compared to wild-type controls (n=3-8, * = p<0.05, **=p<0.01, one-way ANOVA). Mean ± s.e.m. shown. See Table S1 for primer sequences.

Development • Supplementary information

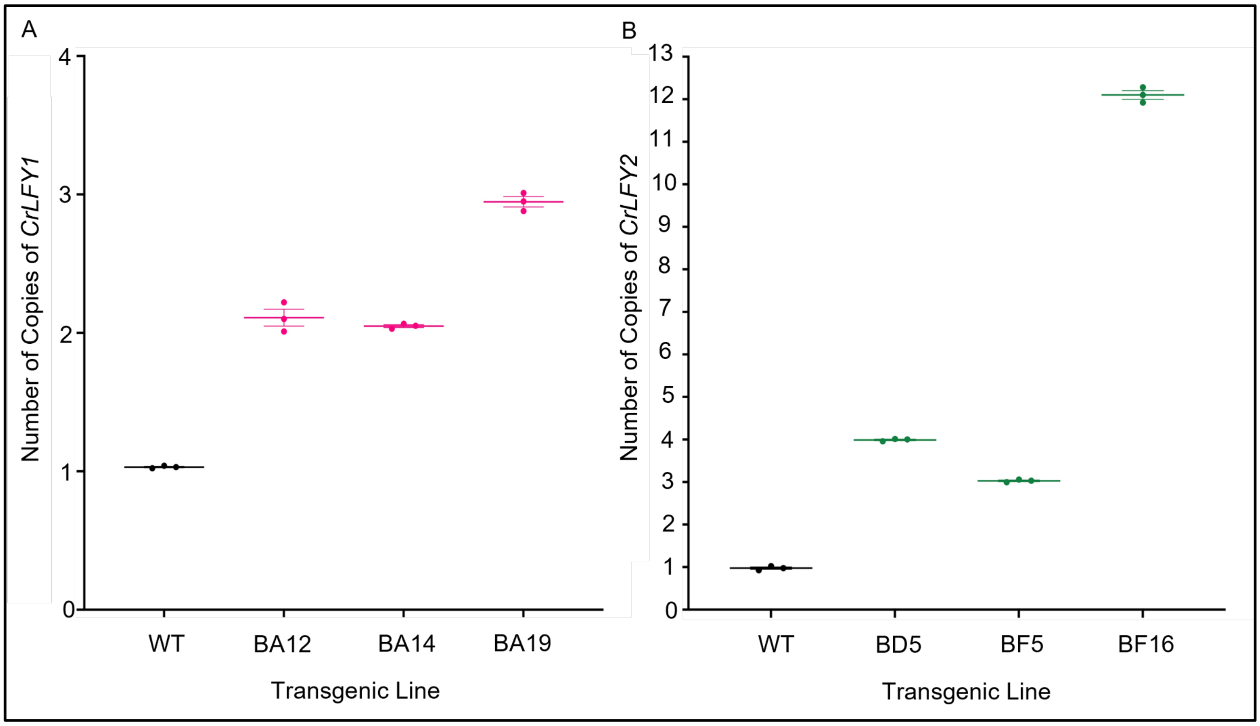

Fig. S2. Insert copy number estimation by digital droplet PCR for the transgenic plant lines characterized in this study.

(A) Number of *35S::CrLFY1* construct insertions in the three transgenic lines used in this study, compared to wild type (WT). Two transgenic lines, BA12 and BA14, contain 2 total copies, or one endogenous and one inserted copy of *CrLFY1*, while BA19 contains 3 total copies (i.e., 2 insertions). (B) Number of copies of *35S::CrLFY2* construct insertions in the three transgenic lines used. transgenic line BD5 contains 4 total copies of *CrLFY2*, or one endogenous and 3 inserted, while BF5 contains 3 total copies, and BF16 contains 12. Mean ± s.e.m. shown.

Development • Supplementary information

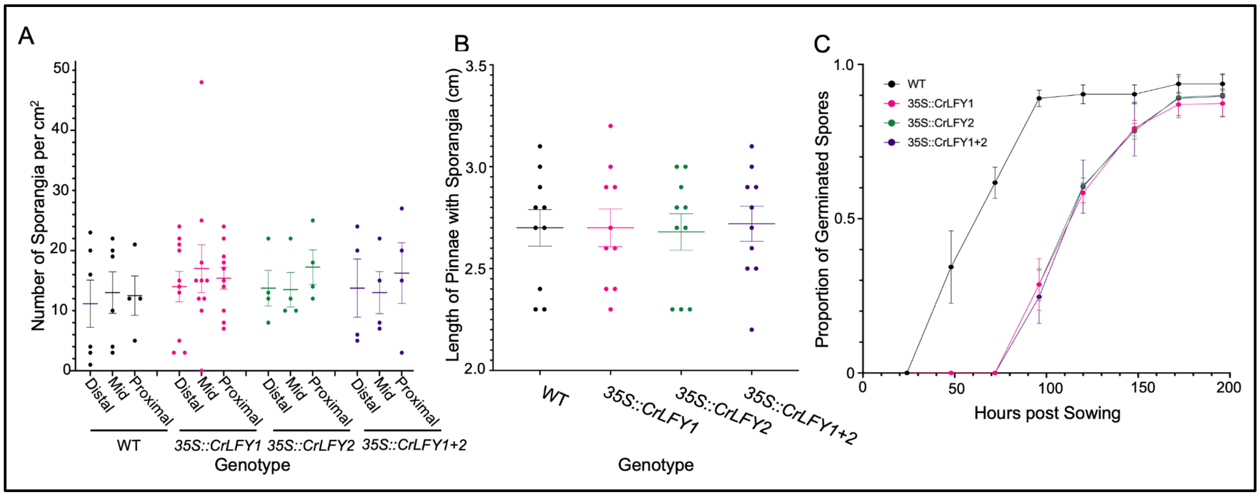

Fig. S3. Number of sporangia, length of sporophyll pinnae, and spore germination success in wild-type (WT) and transgenic *C. richardii*. (A) The average number of sporangia across the tip, middle, and bottom regions of a frond (n=5-10, p=0.44, two-way ANOVA); (B) Length of individual pinnae from sporophylls bearing sporangia (n=10, p=0.99, two-way ANOVA); and (C) Proportion of WT and transgenic spores that germinate 200 hrs. after sowing, out of 50, from 3 independent plates (n=3, p=0.46, two-way ANOVA). Mean values ± s.e.m. shown.

Development • Supplementary information

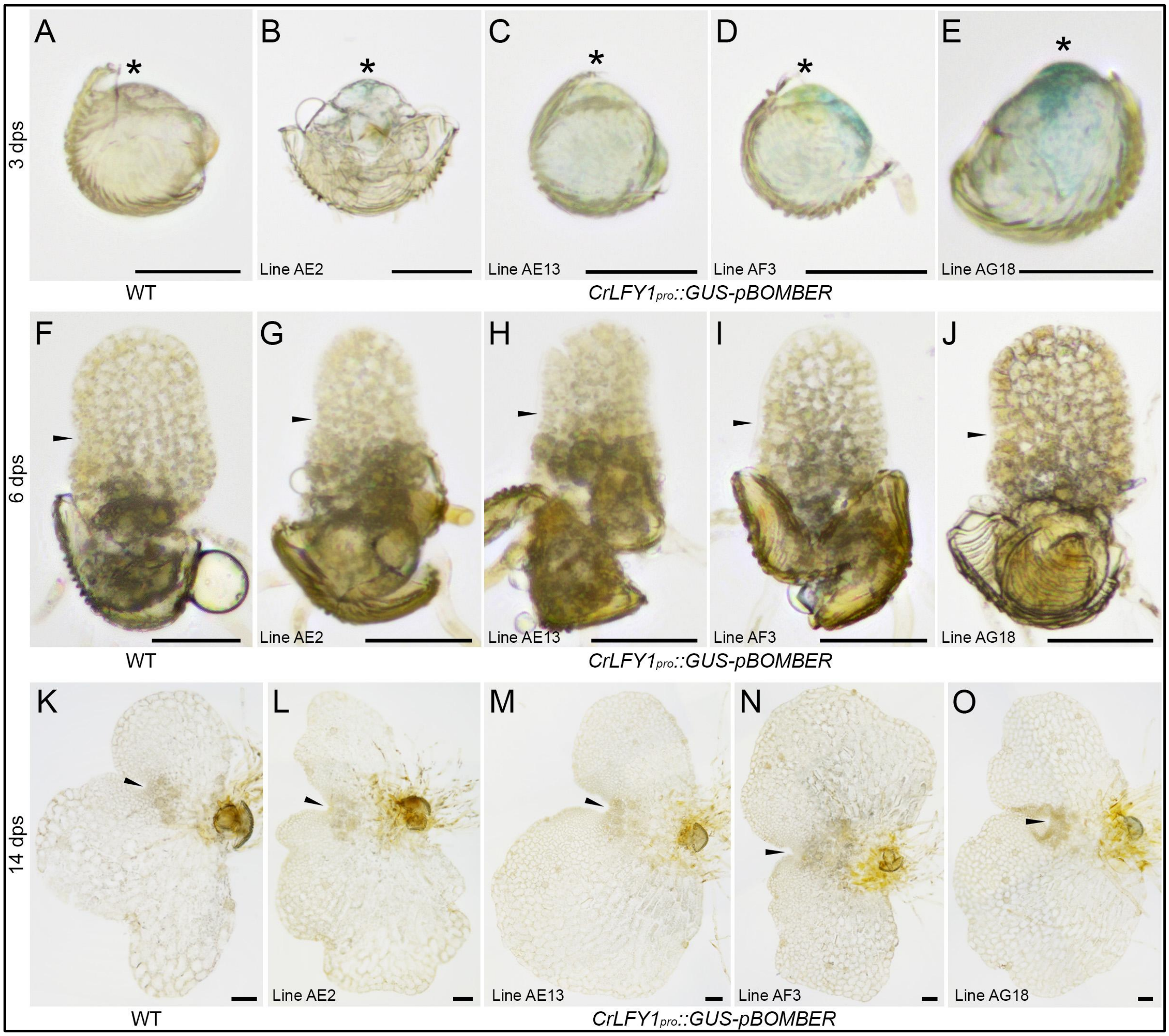

Fig. S4. Additional *CrLFY1$_{pro}$::GUS* gametophyte expression. GUS-stained whole gametophytes at 3dps during spore germination (A-E), 6 dps during notch meristem initiation (F-G) and 14 dps at sexual maturity with a fully active notch meristem (K-O), comparing WT (A, F, K) to four independent transgenic lines carrying *CrLFY1$_{pro}$::GUS* (B-E, G-J, L-O), as previously described (Plackett *et al.* 2018). GUS staining is visible in the apical cell at spore germination, consistent with previous *in situ* localization results. GUS staining was not detected in the notch meristem. Scale bars = 100 μm, asterisks = apical cell, arrowheads = notch meristem.

Development • Supplementary information

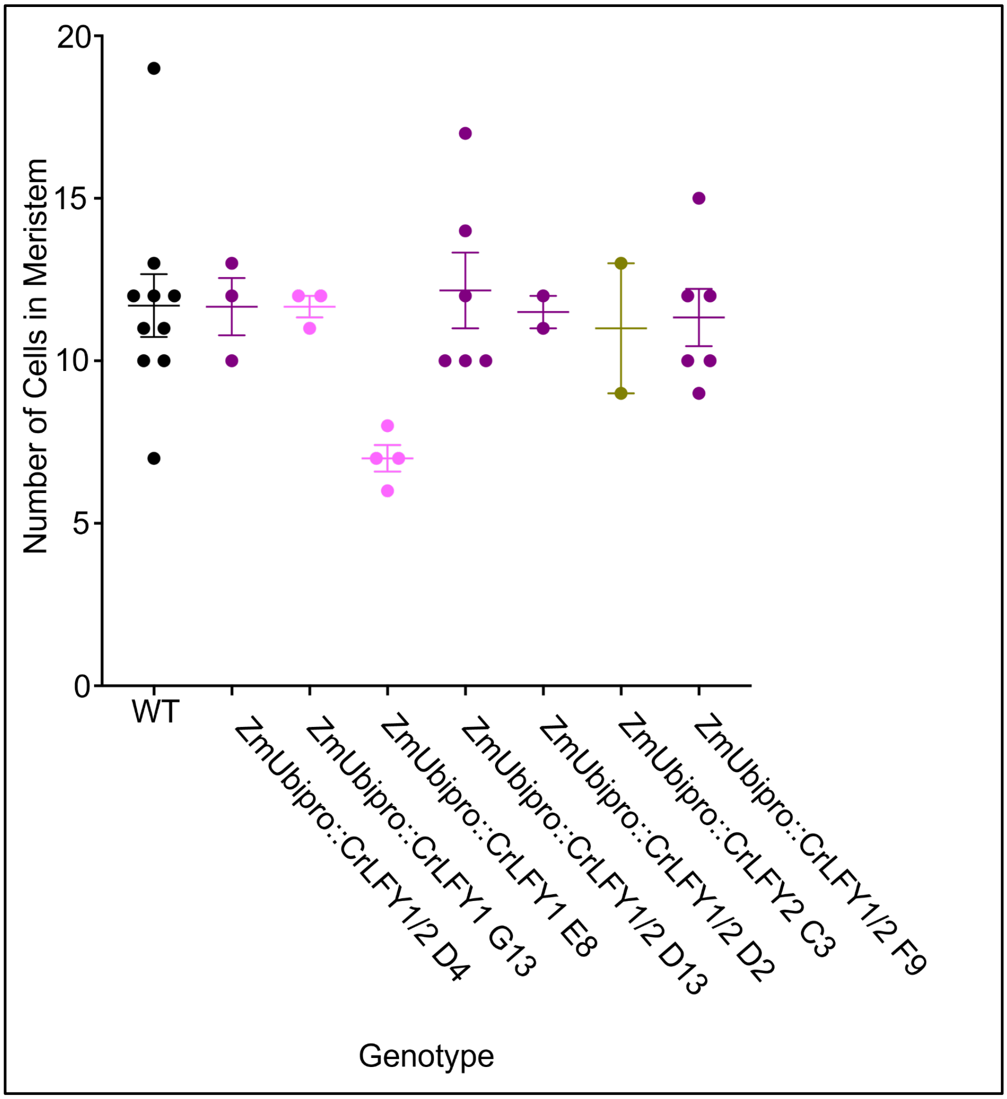

Fig. S5. Quantification of notch meristematic cells in *CrLFY*-RNAi transgenic lines The number of meristematic cells in WT and transgenic *RNAi-CrLFY* (*ZmUbipro::CrLFY1/2)* gametophytes at 13 days post-sowing. Letter and number combinations indicate independent transgenic lines.

Development • Supplementary information

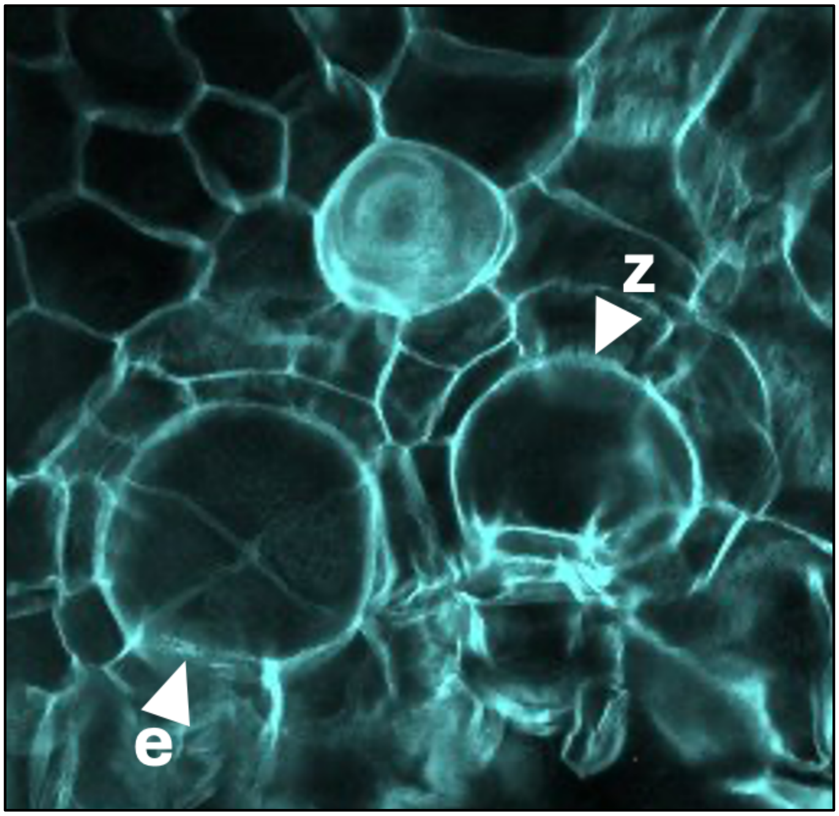

Fig. S6. Example of secondary fertilization in wild-type gametophytes, with multicellular embryo and an arrested zygote. Wild-type gametophytes produce a multicellular embryo (e) within 16 h of fertilization. Representative photo from low-frequency secondary fertilization, with a multicellular embryo (4 cells visible, but likely at 8-cell stage), and a single cell zygote (z) that did not develop into an embryo.

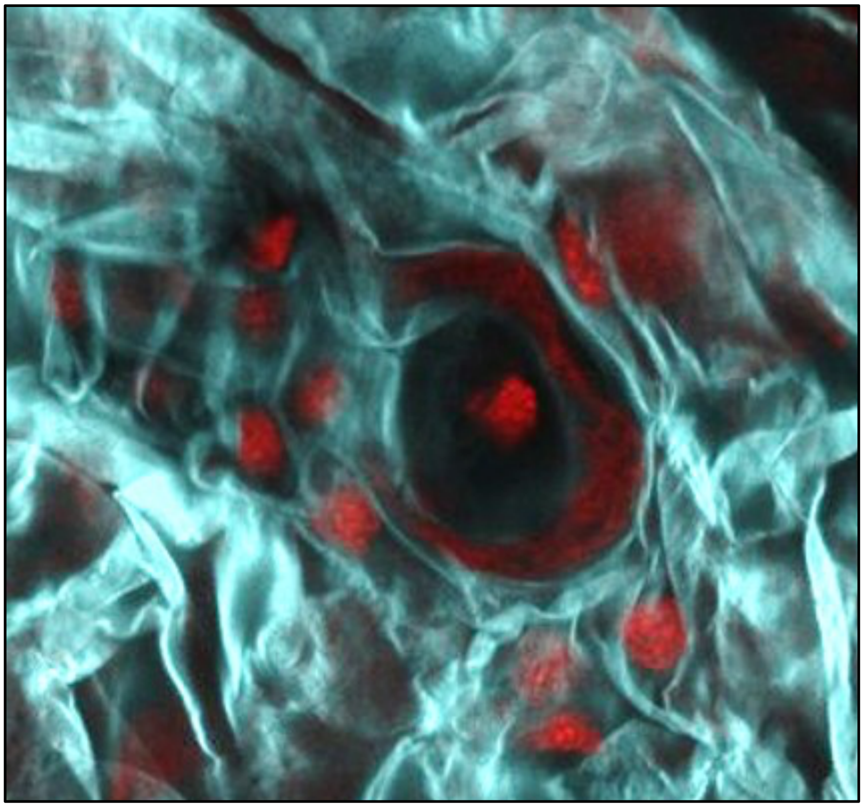

Fig. S7. Decrease in Fern *LFY* expression results in failure of zygote progression. Partial Z-stacks from confocal images of an archegonium in a RNAi-*CrLFY* (*ZmUbipro::CrLFY1/2-i)* gametophyte one day after flooding stained with SR220 (light blue, cell wall) and Propidium Iodide (red, nuclei). The zygote has not progressed beyond the one-cell stage and is representative of all stalled zygotes found in the RNAi-*CrLFY* plants, whereas multicellular embryos are found in wild-type controls at this stage.

Development • Supplementary information

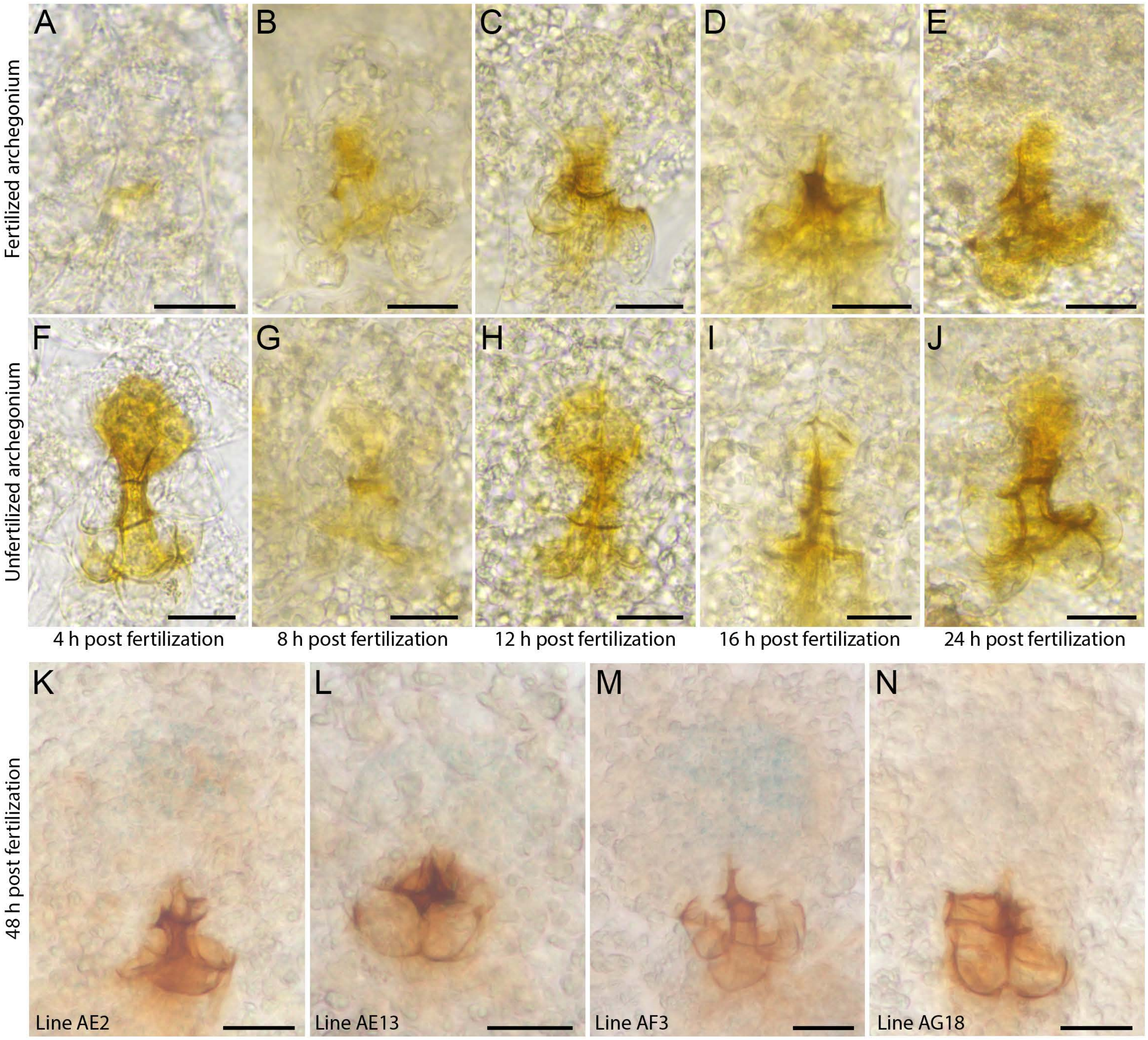

Fig. S8. *CrLFY1$_{pro}$::GUS* expression during zygote and early embryo development. (A-J) *CrLFY1$_{pro}$::GUS* archegonia (line AG18) GUS-stained 4 (A,F), 8 (B,G), 12 (C,H), 16 (D,I) and 24 hours (E,J) after fertilization, showing fertilized archegonia (A-E) compared to unfertilized archegonia (F-J) from the same gametophyte per timepoint. (K-N) The earliest developmental stage post fertilization where GUS staining became visible, comparing four independent transgenic lines carrying *CrLFY1$_{pro}$::GUS*as previously described (Plackett *et al.* 2018). Scale bars = 25 μm.

Table S1. Statistics (*p*-values) for the proportion of multicellular embryos observed up to two weeks after fertilization (flooding) from two-way ANOVA. The proportion of multicellular embryos for each genotype was compared to the proportion of multicellular embryos found in wild type on each day, *n*=36, bold indicates a statistically significant difference.

Sperm Contribution	Construct	Day 2	Day 6	Day 9	Day 10	Day 11	Day 12	Day 13	Day 14
Same genotype	35S::*CrLFY1*	**<0.05**	**<0.01**	0.10	**<0.05**	**<0.01**	**<0.01**	**<0.01**	**<0.01**
	35S::*CrLFY2*	**<0.01**	**<0.01**	0.07	**<0.01**	**<0.001**	**<0.01**	**<0.01**	**<0.05**
	35S::*CrLFY1+2*	**<0.001**	**<0.05**	0.06	**<0.05**	**<0.01**	**<0.01**	**<0.01**	**<0.01**
Transgenic flooding WT	35S::*CrLFY1*	0.39	**<0.01**	0.13	**<0.05**	**<0.001**	**<0.001**	**<0.001**	**<0.001**
	35S::*CrLFY2*	**<0.05**	**<0.01**	0.11	**<0.05**	**<0.001**	**<0.001**	**<0.001**	**<0.01**
	35S::*CrLFY1+2*	0.37	**<0.05**	0.09	**<0.05**	**<0.01**	**<0.01**	**<0.05**	**<0.05**
WT flooding transgenic	35S::*CrLFY1*	0.98	0.070	0.24	0.09	**<0.05**	**<0.05**	**<0.01**	0.07
	35S::*CrLFY2*	0.98	0.25	0.27	0.11	**<0.05**	**<0.05**	**<0.01**	0.07
	35S::*CrLFY1+2*	0.65	0.14	0.23	0.11	**<0.05**	**<0.05**	**<0.05**	0.08
WT flooding transgenic vs Same genotype	35S::*CrLFY1*	0.43	0.17	**<0.05**	**<0.05**	0.57	**<0.05**	**<0.05**	**<0.05**
	35S::*CrLFY2*	0.24	0.03	0.12	**<0.05**	0.06	**<0.05**	**<0.05**	**<0.05**
	35S::*CrLFY1+2*	0.07	0.27	0.33	**<0.05**	**<0.05**	**<0.05**	**<0.05**	**<0.05**

Development • Supplementary information

Table S2. Primers used in this study (qPCR primers from Plackett et al., 2018).

**Primer name**	**Sequence** 5'→ 3'	**Purpose**
qCrLFY1F	ACA AGC ATG CTA TTA TCC ATT GGT	qPCR gene expression
qCrLFY1R	TCA CTG TCC TTG CTC TTC TCT AAA	qPCR gene expression
qCrLFY2F	GAC TCC TTG CTC TAC CTG AAC CTA	qPCR gene expression
qCrLFY2R	CTT CAC CAG GCT CTG TCA CTA TAA	qPCR gene expression
qCrACT1_F	GAG AGA GGC TAC TCT TTC ACA ACC	qPCR reference gene
qCrACT1_R	AGG AAG TTC GTA ACT CTT CTC CAA	qPCR reference gene
qCrTBPb_F	ATG AGC CAG AGC TTT TCC CC	qPCR reference gene
qCrTBPb_R	TTC GTC TCT GAC CTT TGC CC	qPCR reference gene
HygF2	CTTCTACACAGCCATCGGTC	Transgenic validation
HygR	CCGATGGTTTCTACAAAGATCG	Transgenic validation
CrLFY1_F	AAATAGGGCCACCTGGACTC	Transgenic validation
CrLFY1_R	CATTCTTCTTTCCCCTTGCC	Transgenic validation
CrLFY2_F	TGTAGAAGGCACCAGGGAAC	Transgenic validation
CrLFY2_R	TCCCCGTCCTCACCAGGCTC	Transgenic validation
35S_end	AAACCTCCTCGGATTCCATT	Transgenic validation
OCSend	TTAGAATGAACCGAAACCGG	Transgenic validation
CrLFY1_4,707 F	ACCTGGACTCCTGGCTCTAC	ddPCR insert number
CrLFY1_4,874 R	TTTCCCCTTGCCACTTCACC	ddPCR insert number
CrLFY2_1,868 F	ACTGCTGCTCAGAATGGTCCC	ddPCR insert number
CrLFY2_2,070 R	TCTCTGGTCCTGTCATCCCC	ddPCR insert number

Table S3. dataset for graphs in Figs. 1-5.

Available for download at
https://journals.biologists.com/dev/article-lookup/doi/10.1242/dev.204808#supplementary-data

Development • Supplementary information

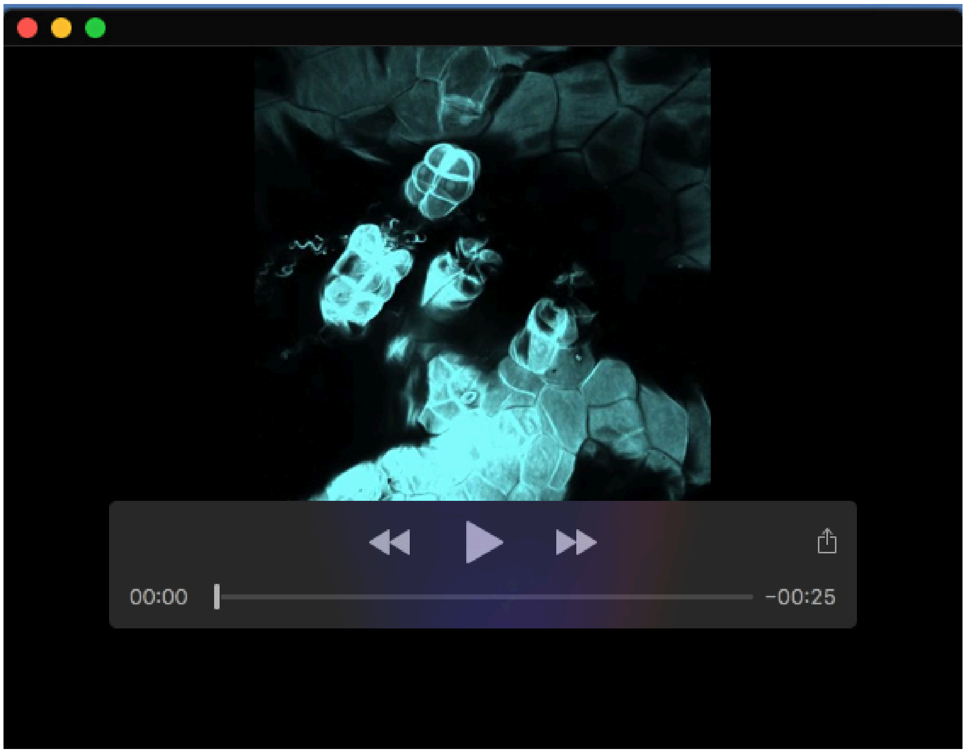

Movie 1. Wild-type archegonium before fertilization. Confocal 3D reconstruction (Z-stack) of a wild-type unfertilized mature gametophyte showing an archegonium (female gametangium) with neck cells and a venter containing a ventral canal cell and an egg cell.

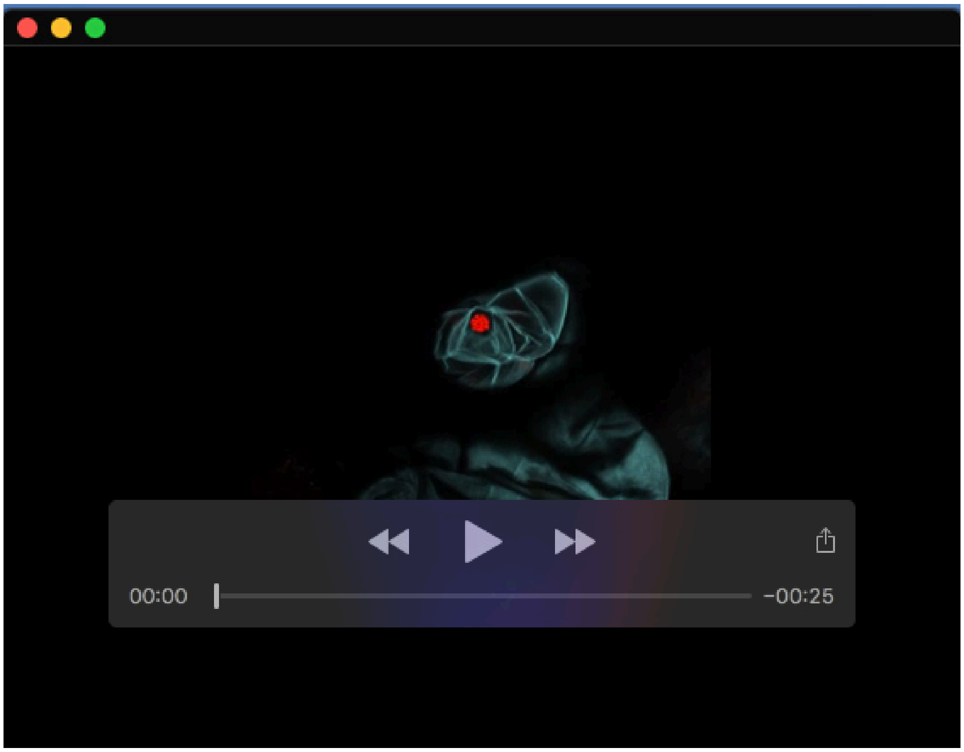

Movie 2. Wild-type zygote. Confocal 3D reconstruction (Z-stack) of a wild-type gametophyte that has undergone fertilization to produce a zygote 2 h after flooding.

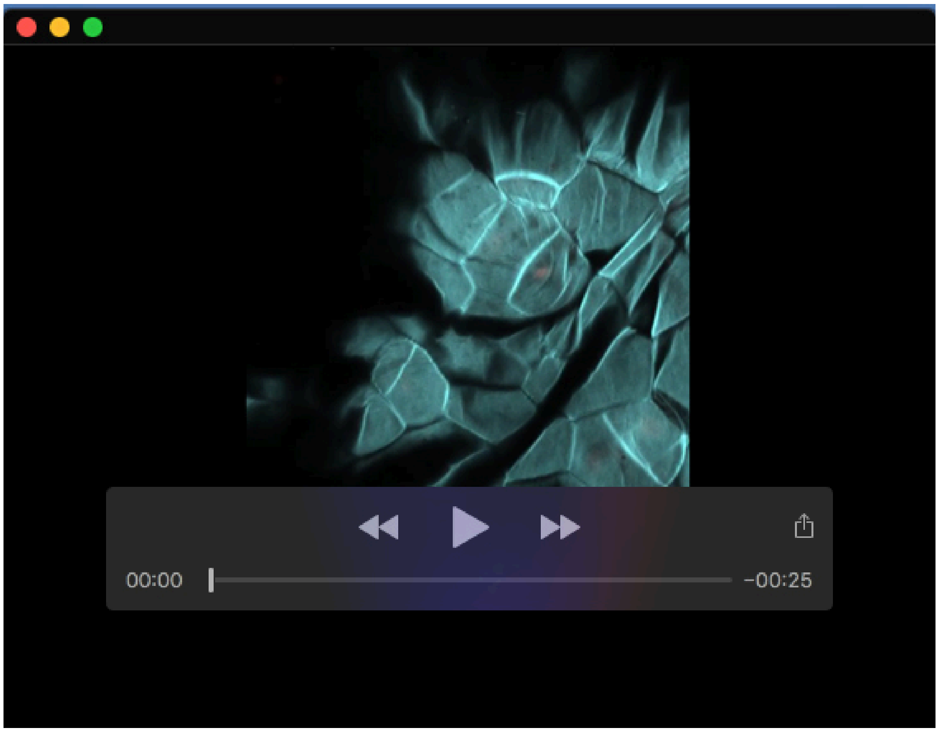

Movie 3. Wild-type multicellular embryo. Confocal 3D reconstruction (Z-stack) of a wild-type gametophyte with a multicellular embryo 16 h after flooding.

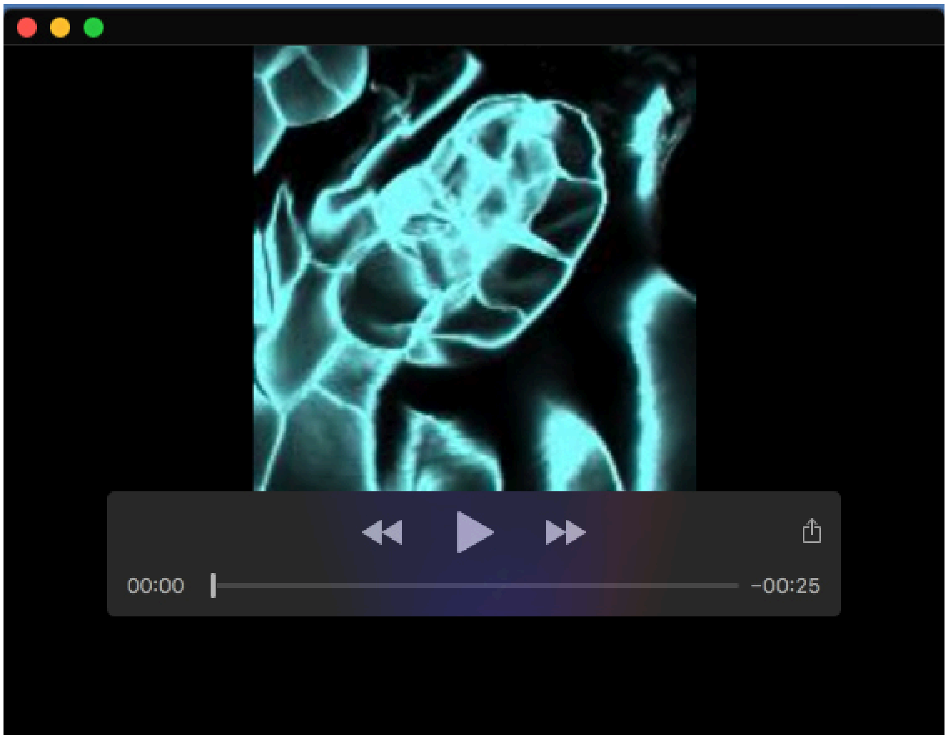

Movie 4. Wild-type unfertilized egg cell after fertilization. Confocal 3D reconstruction (Z-stack) of wild-type gametophyte with an unfertilized egg cell 16 h after flooding.

Development • Supplementary information

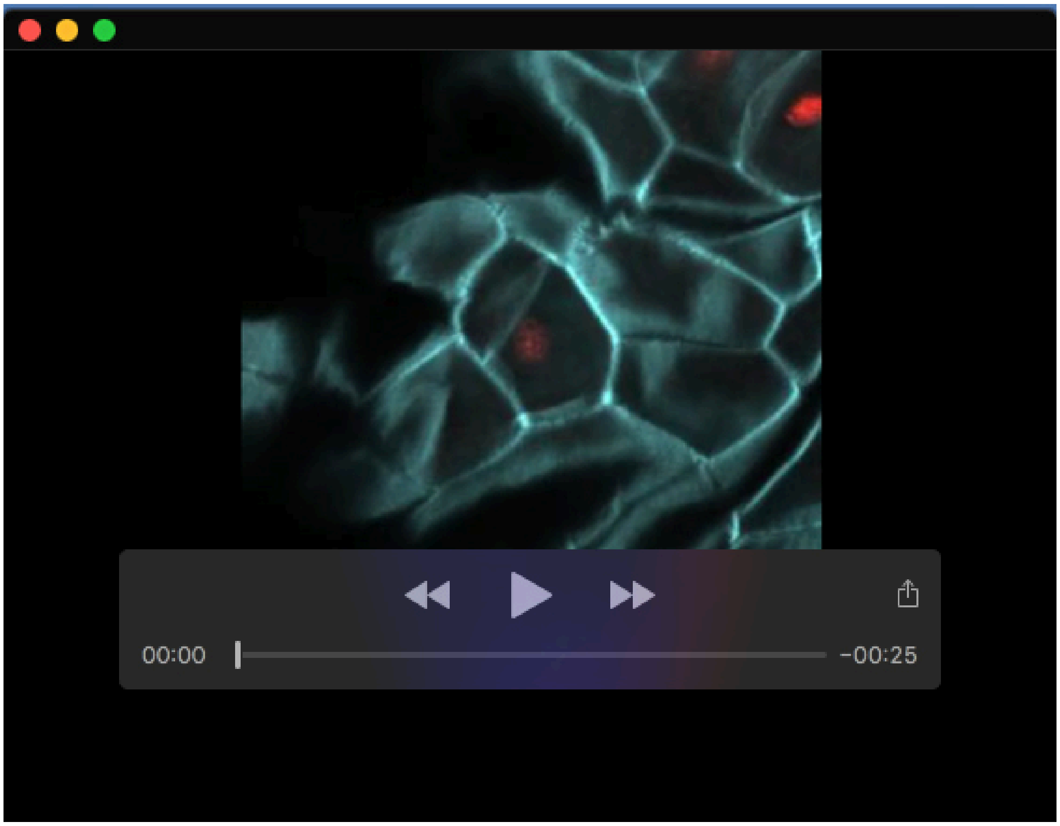

Movie 5. Arrested transgenic zygote. Confocal 3D reconstruction (Z-stack) of transgenic gametophyte (*35S::CrLFY1+2*) exhibiting a single cell with an elongated nucleus 16 h after flooding, interpreted as a zygote arrested before the first cell division.
